# Association of a Single-Item Self-Rated Diet Construct With Diet Quality Measured With the Alternate Healthy Eating Index

**DOI:** 10.3389/fnut.2021.646694

**Published:** 2021-05-05

**Authors:** Cristina M. Gago, Andrea Lopez-Cepero, June O'Neill, Martha Tamez, Katherine Tucker, José F. Rodríguez Orengo, Josiemer Mattei

**Affiliations:** ^1^Department of Nutrition, Harvard T.H. Chan School of Public Health, Boston, MA, United States; ^2^Department of Biomedical & Nutritional Sciences, Zuckerberg College of Health Sciences, University of Massachusetts Lowell, Lowell, MA, United States; ^3^School of Medicine, University of Puerto Rico, Medical Sciences Campus, San Juan, PR, United States; ^4^FDI Clinical Research, San Juan, PR, United States

**Keywords:** diet quality, self-rated diet quality, alternate healthy eating index, underrepresented and minority groups, Puerto Rico

## Abstract

**Background:** A single-item self-rated diet measure (SRD) may provide a quick, low-burden screener. However, assessment of its validity is limited. This study aimed to evaluate the association of an SRD construct with measured diet quality among adults in Puerto Rico (PR).

**Methodology:** Participants (30–75 years old; *n* = 247) of the PR Assessment of Diet, Lifestyle, and Diseases (PRADLAD) cross-sectional study reported SRD with a single question (“How would you describe your current dietary habits and diet quality?”) with a five-point scale: excellent to poor. More complete diet quality was calculated using the Alternate Healthy Eating Index-2010 (AHEI), with 11 food and nutrient components assessed by the food frequency questionnaire. Multivariable general linear models were used to test associations between SRD with AHEI and its components. Associations were also tested between recall SRD in youth and current AHEI.

**Results:** Most participants (35.2%) self-rated diet as “good,” 13.8% as “excellent,” and 4.1% as “poor,” with the remainder split between middle scale points. SRD was not significantly associated with AHEI, although participants with “excellent” vs. “poor” SRD had marginally higher AHEI (*P* = 0.07). SRD was significantly associated with higher fruit intake (*P* = 0.02) and marginally associated with intakes of vegetables (*P* = 0.07) and long-chain fatty acids (*P* = 0.07). Unexpectedly, AHEI was significantly higher among those reporting “poor” SRD in young adulthood (*P* = 0.01) or childhood (*P* = 0.05).

**Conclusions:** SRD may capture current diet quality at extreme intakes. Larger studies should confirm these findings and replicate them in other underrepresented populations. Further research should clarify the inverse associations between adult AHEI and earlier reported SRD.

## Introduction

Extensive research exists on the relationship between perception and awareness of dietary quality in relation to diet intake and chronic disease risk (1, 2). However, this research tends to be restricted to North America and Europe (1, 2). Most studies focus on multi-item measures of diet quality, which are often expensive, time-consuming, and resource intensive (3). In contrast, a single-item self-rated diet measure (SRD) may offer epidemiologists and clinical practitioners a rapid and cost-effective means of differentiating individuals at extreme diet quality intake (e.g., “excellent” vs. “poor” diet) (4), for risk stratification and resource allocation.

Construct validity of single-item measures may be established by evaluating associations with energy balance, self-reported food-related behaviors, dietary intake biomarkers, and clinical measures related to diet (5). However, there are fewer studies of single-item measures of diet perception in comparison with objective diet quality measures. Of those that exist, few are derived from samples outside urban centers of Europe and North America (4–6), highlighting a wide gap in validation of this construct within the unique cultural setting of diverse populations. Previous authors have called attention to the lack of evidence regarding the impact of sociocultural factors—such as language and country of origin—on diet quality awareness and reporting (5). Testing this measure in unique cultural contexts is necessary, as self-reported health measures have consistently been shown to be susceptible to cultural bias, especially among Latinos and other groups (7). Even after adjustment for socioeconomic status, differences in self-reported health are still evident, predominantly among US Latino and Asian populations, leading previous researchers to propose that these differences may be explained by culture and language (7).

Responding to this gap in the literature, our study aimed to evaluate the construct validity of a single-item SRD question and its association with measured diet quality among adults in Puerto Rico (PR). This setting is especially important as Puerto Ricans exhibit documented suboptimal dietary intake and associated risk of cardiometabolic diseases (8–10). We hypothesized that higher SRD would be associated with significantly higher measured diet quality.

Secondarily, we aimed to evaluate whether the single-item recall measure of diet quality in earlier life stages (e.g., young adulthood and childhood) is associated with current measured diet quality in adulthood. Although there is a large body of research regarding the validity of parental perceptions of child diet (11–15), less is known about the validity of SRD in one's childhood and young adulthood. Individuals may experience different trajectories that predict either stable (healthy or unhealthy) or changed health-related behaviors from adolescence to adulthood (16). Taking a life course approach to chronic disease epidemiology (17), researchers are highlighting the importance and need for extensive research on the relationship between time and timing on both health habit formation and related chronic disease development (17). Thus, we posit that internalized perceptions of previous diet may be associated with current diet. If such a connection is supported, further research on the mechanism underlying the intersection of former and current diet perception would be warranted.

## Methods

### Study Design

The Puerto Rico Assessment of Diet, Lifestyle, and Diseases (PRADLAD) study is cross-sectional in design. Details of the study design and methodology have been previously published (18). Adult respondents living in PR (*n* = 380) were surveyed while visiting or awaiting care at three clinics in the San Juan metro area, *via* convenience sampling in 2015. Eligibility for the study included residency in PR for at least 10 consecutive months (from recruitment date), aged 30–75 years, and capability of answering survey questions independently. Participants provided written informed consent. The study was approved by the Institutional Review Boards at Harvard T.H. Chan School of Public Health, Ponce Health Sciences University in PR, University of Massachusetts Lowell, and Northeastern University. Written informed consent was obtained from all the participants.

### Self-Rated Diet Quality

Respondents were asked to self-rate their dietary habits and diet quality on a five-point Likert scale (“excellent,” “very good,” “good,” “fair,” and “poor”) using the question “How would you describe your current dietary habits and diet quality?” The same question was asked separately in reference to diet quality “when you were a child (until 18 years old)” and as a “young adult (between 18 and 30 years old).” To improve power across categories, a variable with three categories was created and tested in sensitivity analysis: “excellent or very good,” “good,” or “fair or poor.”

### Measured Diet Quality

Dietary intake was assessed using a semiquantitative culturally adapted food frequency questionnaire (FFQ) (19), which has been validated for use among adults in PR. Although the FFQ is self-reported, it has been consistently cited as a validated measure of intake (20, 21). Furthermore, previous studies support the tool's validity and reliability with respect to energy and nutrient estimates in Puerto Ricans (19). Foods and nutrients were analyzed using the Minnesota Nutrient Data System (version 5.0_35). Diet quality was defined using the Alternate Healthy Eating Index-2010 (AHEI) (22). A continuous AHEI component score (ranging from 0 to 10) was calculated for each of the 11 food groups or nutrients: vegetables, fruits, nuts and legumes, whole grains, red meats, sugar-sweetened beverages (SSBs), alcohol, polyunsaturated fatty acids (PUFA), trans fats, long-chain fatty acids (LCFA), and sodium. Scores were summed, and the total AHEI score ranged from 0 to 110, with a low score indicating low nutritional quality and a high score indicating high nutritional quality.

### Data Collection

Trained bilingual (Spanish/English) interviewers collected information on general demographics (e.g., sex and ethnicity), socioeconomic factors (e.g., educational attainment, household income, marital status, area of residency), and health behaviors [smoking status (current, never, past), physical activity, and sleep duration]. These three health behaviors were selected for inclusion in the models based on evidence of association with both self-rated health status and nutritional intake (23–27). Physical activity was assessed as a continuous measure, *via* a modified Paffenbarger questionnaire of the Harvard Alumni Activity Survey (28); responses were categorized across three classifications (e.g., sedentary, light, and moderate or vigorous) based on the summation of average daily activity and multiplication by factors with similar rates of oxygen usage (29). Furthermore, participants were asked to report their average duration of sleep per night; “healthy sleep” was defined as 7 to 8 h per night, and “unhealthy sleep” as outside this range (30). The interviewer measured the participant's waist circumference using a Gulick measuring tape and following standard protocols (18).

### Statistical Analysis

PRADLAD participants (*n* = 380) completed a main questionnaire. Of these, 247 (65%) who completed both the FFQ and provided a response to the SRD question were included in the analysis. Sample characteristics were contrasted by SRD category using chi-square tests or Fisher's exact test for categorical variables and ANOVA for continuous variables. Missingness was due to standard exclusions for absence of FFQ data, incomplete FFQ data, and outlying energy intake suggestive of misreporting, or unreliable data (18).

We calculated Spearman's Rho to assess the direction and magnitude of correlation shared by continuous SRD (in adulthood, young adulthood, and childhood), and continuous total AHEI score, and each of the 11 AHEI components. Secondarily, ANOVA was used to assess differences in unadjusted mean AHEI across SRD categories. Applied multivariable general linear regression model (GLM) was used to evaluate relationships between mean AHEI score (as a continuous variable) and SRD in adulthood and, as exploratory analyses, recall of SRD in young adulthood and childhood.

Finally, GLM was used to assess relationships between each continuous AHEI component score (11 different models) and adulthood SRD (both categorically and continuously). All regression models were adjusted for sex, ethnicity, educational attainment, household income, marital status, residency (rural vs. urban), sleep quantity, smoking status, and physical activity level. Further adjustment for nutritional awareness, central obesity, or total energy intake did not substantially change the model and was omitted in favor of a more parsimonious model. The impact of missingness in covariates was tested. A “missing” category was created and modeled for three items [e.g., physical activity (*n* = 95), sleep quantity (*n* = 14), and income (*n* = 32)] to capture observations when covariate data were missing; models with and without missingness categories presented similar results, so imputation was deemed unnecessary.

As a secondary analysis, we examined the association between SRD and continuous waist circumference (31) in a subset of 202 participants that had this measure available. To confirm consistency in the results, the three-category SRD scale was used as the predictor for all analyses. To calculate sensitivity and specificity, binomial variables were created using the median AHEI as the “truth” (range: 35.9–96.9; “positive” ≥58.7), comparing proportions to the current SRD item responses as the “test” (“positive” = “good” + “very good” + “excellent”). These metrics were tested as an internal reference to substantiate the findings of the GLM rather than to validate the SRD item as a clinical diagnostic tool. All analyses were conducted with SAS software version 9.4 (SAS Institute Inc., Cary, NC). We considered significant differences to be at two-tailed at *P* ≤ 0.05.

## Results

### Sample Characteristics

The mean (SD) age of our sample was 51.5 (10.9) years (Table 1). Most participants were identified as female (70%), of Puerto Rican ethnicity (79%), never-smokers (71%), living in an urban community (90%), and had at least some college education (62%). Approximately half of the participants reported earning < $10,000 per year (50%), being married or living with a partner (43%), and having unhealthy sleep quantity (44%). Only area of residence and smoking were found to differ significantly by self-rated diet in adulthood.

**Table 1:** Characteristics of adults living in Puerto Rico, by self-reported diet quality on a five-point scale, 2015 (*n* = 247).

		**How would you describe your current diet? (i.e., SRD)**					***P*-value^**D**^**
	**Overall (*n* = 247)**	**Excellent (*n* = 34; 13.8%)**	**Very good (*n* = 58; 23.5%)**	**Good (*n* = 87; 35.2%)**	**Fair (*n* = 58; 23.5%)**	**Poor (*n* = 10; 4.1%)**	
Age (years)^A^	51.5 (10.9)	52.5 (8.8)	50.5 (11.8)	52.03 (11.1)	51.7 (10.9)	48.3 (12.7)	0.77
Female (vs. other)	69.2	67.7	70.7	65.5	74.1	70.0	0.86
Puerto Rican (vs. other)	79.4	70.6	84.5	79.3	81.0	70.0	0.53
Education							0.83
<8th grade	11.3	17.7	12.1	9.20	10.3	10.0	
High school or GED	26.3	17.7	24.1	26.4	32.8	30.0	
Some college or more	61.9	64.7	63.8	64.4	55.2	60.0	
Income							0.31
$0–10,000	49.8	8.8	12.1	13.8	15.5	10.0	
$10,001–$20,000	20.2	61.8	44.8	47.1	50.0	60.0	
>$20,000	17.0	5.9	25.9	27.6	13.8	10.0	
With partner (vs. other)	43.3	38.2	41.4	42.5	50.0	40.0	0.82
Urban residency (vs. rural)	89.9	94.1	96.6	85.1	86.2	100	0.05*
Sleep duration^B^							0.15
Healthy 7–8 h	50.6	40.0	50.0	51.7	51.7	50.0	
Unhealthy <7 or >8 h	43.7	50.0	36.2	41.4	50.0	50.0	
Smoking status							0.02*
Current	17.4	17.7	10.3	16.1	24.1	30.0	
Never	70.5	64.7	86.2	67.8	67.2	40.0	
Past	11.7	17.7	3.5	16.1	6.9	30.0	
Physical activity level^**C**^							0.40
Light	29.6	35.3	24.1	29.9	34.5	10.0	
Moderate	22.7	20.6	22.4	18.4	29.3	30.0	
Heavy	38.5	35.3	46.6	43.7	22.4	50.0	
Waist circumference (cm) ^A^	97.8 (17.2)	97.5 (11.9)	93.4 (19.9)	99.6 (19.9)	100.2 (16.0)	93.4 (18.8)	0.27

*Shown as percent of sample (n = 247). Sample sizes for individual variables may not add up to 100% due to missingness*.

*SRD, self-rated diet (possible range = 1–5, “poor” to “excellent” diet quality)*.

A *Shown as unadjusted mean (SD)*.

B *“Healthy sleep” was assigned to those who reported sleeping an average of 7 to 8 h per day and “unhealthy sleep” to those outside this range*.

C *Physical activity was categorized across three levels of “sedentary,” “light,” and “moderate,” based on the results of the Paffenbarger questionnaire of the Harvard Alumni Activity Survey*.

D *Significant differences by self-rated diet (five-point categorical variable) were determined from ANOVA or chi-squared test and confirmed with Fisher's exact test*.

Significance shown as *P ≤ 0.05; **P ≤ 0.01; ***P ≤ 0.001.

### AHEI by Adulthood SRD

Mean adjusted total AHEI score did not differ significantly by SRD (Table 2). After adjusting for potential confounders, mean AHEI tended to be higher among those with higher SRD: 61.3 (4.74), 58.5 (4.88), 58.2 (4.52), 58.1 (4.54), and 53.8 (5.76) from “excellent” to “poor” SRD, respectively (Table 2). Participants with “excellent” SRD in adulthood had marginally higher AHEI—by a mean estimated 7.46 units (95% CI: −0.53, 15.5; *P* = 0.07; Table 2)—compared with those with “poor” SRD. Similar patterns were observed with a three-point SRD scale (Supplementary Table 1). Fruit was the only AHEI component found to be significantly correlated with SRD in adulthood (*P* = 0.03; Table 3). Similar patterns were observed with a three-point SRD scale (Supplementary Table 2). In secondary analysis, after adjusting for other potential confounders, SRD in adulthood was not significantly associated with continuous waist circumference [adjusted mean score (SE): 91.2 (7.89) vs. 85.9 (9.37) for those with “excellent” vs. “poor” SRD] (Supplementary Table 3).

**Table 2:** Adjusted mean Alternate Healthy Eating Index score by self-rated diet as a continuous and categorical variable at three life stages (adulthood, young adulthood, childhood) on a five-point scale (*n* = 247).

**Life stage**	**SRD**	**Adjusted mean AHEI (SE)^**A**^**	**β (95% CI)^**B**^**	***P*-value^**B**^**
Adulthood (current)	Excellent (*n* = 34, 13.8%)	61.3 (4.74)	7.46 (−0.53, 15.5)	0.07
	Very good (*n* = 58, 23.5%)	58.5 (4.88)	4.70 (−3.02, 12.4)	0.23
	Good (*n* = 87, 35.2%)	58.2 (4.52)	4.39 (−3.07, 11.8)	0.25
	Fair (*n* = 58, 23.5%)	58.1 (4.54)	4.30 (−3.40, 12.0)	0.27
	Poor (*n* = 10, 4.1%)	53.8 (5.76)	Ref	Ref
Young adulthood	Excellent (*n* = 31, 12.6%)	55.7 (4.69)	−6.46 (−12.5, −0.43)	0.04^*^
(18–30 years old)	Very good (*n* = 43, 17.4%)	53.6 (4.66)	−8.55 (−14.2, −2.92)	0.003^**^
	Good (*n* = 83, 33.6%)	58.4 (4.44)	−3.74 (−8.81, 1.32)	0.15
	Fair (*n* = 65, 26.3%)	60.1 (4.44)	−2.05 (−7.25, 3.15)	0.44
	Poor (*n* = 25, 10.1%)	62.2 (4.75)	Ref	Ref
Childhood (<18 years old)	Excellent (*n* = 56, 22.7%)	59.1 (4.56)	0.65 (−4.90, 6.20)	0.82
	Very good (*n* = 50, 20.2%)	56.7 (4.55)	−1.80 (−7.43, 3.83)	0.53
	Good (*n* = 75, 30.4%)	56.2 (4.51)	−2.29 (−7.57, 2.99)	0.39
	Fair (*n* = 43, 17.4%)	62.6 (4.54)	4.10 (−1.66, 9.86)	0.16
	Poor (*n* = 23, 9.31%)	58.5 (5.11)	Ref	Ref

*AHEI, Alternate Healthy Eating Index (possible range = 0.0–110.0, observed range = 35.9–97.0, low to high diet quality). SRD, self-rated diet (possible range = 1–5, “poor,” “fair,” “good,” “very good,” “excellent” diet quality)*.

*All regression models adjusted for age, sex, ethnicity (Puerto Rican vs. other), education (<8th grade, high school or GED, or some college or more), income ($0–10,000; $10,001–20,000; >$20,000), residency (i.e., urban vs. rural), marital status (married or with a partner vs. other), sleep quantity (i.e., healthy, extreme, or missing), smoking status (i.e., current, never, past), and physical activity (i.e., sedentary, light, moderate, or missing)*.

A *Shown as the adjusted mean (SE). Significance of differences in unadjusted mean values across SRD categories was also examined using ANOVA; differences were not found to be significant*.

B *SRD was modeled as a five-point categorical exposure (i.e., “poor,” “fair,” “good,” “very good,” “excellent”). The reference category was ‘poor.' GLM was used to evaluate the relationship between SRD in relation to continuous overall AHEI score; each model includes one predictor variable (i.e., SRD from current day, young adulthood, and childhood). Beta estimates can be interpreted as the difference in mean adjusted AHEI score between a given SRD category and the reference (“poor”)*.

*Significance shown as*

* *P ≤ 0.05;*

** P ≤ 0.01; ***P ≤ 0.001.

**Table 3:** Correlation between Alternate Healthy Eating Index scores and self-rated current diet quality at three life stages (adulthood, young adulthood, childhood) on a five-point scale (*n* = 247).

		**Correlation with self-rated diet quality: Rho (*****P*****-value)**		
**Scores**	**Mean (SD) [range]^**A**^ (*n* = 247)**	**Adulthood**	**Young adulthood**	**Childhood**
AHEI	60.1 (11.4) [35.9–97.0]	0.113 (0.08)	−0.214 (0.07)	−0.024 (0.70)
Vegetables	6.09 (2.90) [0.59–10]	0.112 (0.08)	−0.114 (0.07)	−0.086 (0.18)
Fruits	2.95 (2.60) [0.01–10]	0.137 (0.03)^*^	−0.043 (0.50)	0.047 (0.47)
Nuts and legumes	6.39 (3.22) [0.00–10.0]	0.034 (0.59)	0.087 (0.17)	−0.017 (0.78)
Whole grains	5.36 (3.54) [0.00–10.0]	0.062 (0.33)	−0.135 (0.03)	−0.045 (0.48)
Red/processed meats	4.40 (3.46) [0.00–10.0]	0.002 (0.98)	−0.178 (0.005)^**^	−0.064 (0.32)
SSB	1.64 (3.03) [0.00–10.0]	−0.042 (0.51)	−0.281 (<0.001)^***^	−0.031 (0.63)
Alcohol	6.25 (1.79) [0.00–10.0]	0.026 (0.68)	0.102 (0.11)	0.027 (0.67)
PUFA	6.12 (2.21) [0.95–10]	0.072 (0.263)	−0.006 (0.93)	0.037 (0.57)
Trans fats	7.92 (1.32) [1.66–10]	0.031 (0.62)	−0.152 (0.02)^*^	−0.010 (0.87)
LCFA	6.83 (2.52) [1.54–10]	0.094 (0.14)	0.025 (0.69)	0.024 (0.71)
Sodium	6.12 (3.21) [0–10]	−0.027 (0.68)	−0.113 (0.08)	0.063 (0.32)

*AHEI, Alternate Healthy Eating Index (possible range for overall score = 0.0–110.0, observed range = 35.9–97.0, low to high diet quality; possible range for component scores = 0.0–10.0); SRD, self-rated diet (possible range = 1–5, “poor” to “excellent” diet quality); LCFA, long-chain fatty acid*.

*Significance shown as*

* *P ≤ 0.05;*

** *P ≤ 0.01;*

*** *P ≤ 0.001*.

A *Shown is the unadjusted mean AHEI score (SD) [range]*.

*^B^Shown is Spearman's Rho (P-value), interpreted as the direction and magnitude of correlation shared by SRD (five-point; predictor) and continuous AHEI component scores (outcome)*.

### Component Score by SRD

Two AHEI component scores were significantly different by extreme SRD category (“excellent” vs. “poor”): intakes of fruit and LCFA [adjusted mean score (SE): 4.00 (1.07) vs. 1.99 (1.30); 7.99 (1.07) vs. 5.77 (1.30), respectively; Table 4]; similar results were observed with a three-point SRD scale (Supplementary Table 4). Trend analysis for mean AHEI score across the SRD scale category was only significant for fruit consumption; for every 1-unit increase in SRD category, AHEI fruit component score increased by a mean estimated 0.364 units (CI = 0.061, 0.667 *P* = 0.02).

**Table 4:** Adjusted means of Alternate Healthy Eating Index components by self-rated diet quality (*n* = 247).

		**How would you describe your current diet?**					**β (95% CI)^**C**^**	***P*-+value^**C**^**
**AHEI components**	**Overall^**A**^ (*n* = 247)**	**Excellent^**B**^ (*n* = 34; 13.8%)**	**Very good^**B**^ (*n* = 58; 23.5%)**	**Good^**B**^ (*n* = 87; 35.2%)**	**Fair^**B**^ (*n* = 58; 23.5%)**	**Poor^**B**^ (*n* = 10; 4.1%)**		
Vegetables	6.09 (2.90)	7.01 (1.23)	7.34 (1.27)	6.68 (1.17)	6.46 (1.18)	5.46 (1.49)	0.324 (−0.024, 0.673)	0.07
Fruits	2.95 (2.60)	4.00 (1.07)*	3.17 (1.10)	2.93 (1.02)	2.89 (1.02)	1.99 (1.30)	0.364 (0.061, 0.667)	0.02*
Nuts and legumes	6.39 (3.22)	5.58 (1.42)	5.79 (1.32)	6.46 (1.32)	5.29 (1.68)	6.91 (1.38)	0.105 (−0.288, 0.499)	0.60
Whole grains	5.36 (3.54)	3.74 (1.54)	3.48 (1.42)	2.74 (1.43)	3.82 (1.81)	3.45 (1.49)	0.18 (−0.246, 0.599)	0.41
Red meats	4.40 (3.46)	2.63 (1.45)	2.28 (1.49)	3.12 (1.38)	2.76 (1.39)	2.17 (1.76)	−0.076 (−0.487, 0.334)	0.72
SSB	1.64 (3.03)	1.80 (1.34)	1.57 (1.24)	2.49 (1.25)	2.43 (1.58)	1.82 (1.30)	−0.195 (−0.564, 0.175)	0.30
Alcohol	6.25 (1.79)	8.05 (0.720)	7.38 (0.666)	7.68 (0.669)	8.03 (0.849)	7.88 (0.699)	0.093 (−0.107, 0.292)	0.36
PUFA	6.12 (2.21)	6.54 (0.960)	6.86 (0.889)	6.81 (0.892)	5.86 (1.12)	7.27 (0.933)	0.135 (−0.130, 0.400)	0.32
Trans fats	7.92 (1.32)	7.77 (0.548)	7.75 (0.507)	7.71 (0.509)	7.55 (0.646)	7.92 (0.533)	0.067 (−0.083, 0.218)	0.38
LCFA	6.83 (2.52)	7.99 (1.07)*	7.29 (1.10)*	7.51 (1.02)	7.23 (1.02)	5.77 (1.30)	0.281 (−0.022, 0.585)	0.07
Sodium	6.11 (3.21)	4.42 (1.37)	4.98 (1.41)	5.14 (1.31)	4.91 (1.31)	5.47 (1.66)	−0.157 (−0.544, 0.230)	0.42

*AHEI, Alternate Healthy Eating Index (possible range for component scores = 0.0–10.0); SRD, self-rated diet (possible range = 1–5, “poor” to “excellent” diet quality); PUFA, polyunsaturated fatty acids; LCFA, long-chain fatty acids*.

*All regression models adjusted for age, sex, ethnicity, education, income, residency (i.e., urban vs. rural), marital status, sleep quantity (i.e., healthy vs. extreme), smoking status (i.e., current, never, past), and physical activity (i.e., sedentary, light, and moderate or vigorous)*.

*^14^Shown as the unadjusted mean AHEI score (SD) by categorical SRD*.

B SRD was modeled via GLM as a categorical exposure [i.e., 1 (“poor”) to 5 (“excellent”)]; only associations found to be significant are reported. Shown as the adjusted mean AHEI score (SE) by categorical SRD. Significance shown as *P ≤ 0.05.

C *SRD was modeled via GLM as a continuous exposure [i.e., 1 (“poor”) to 5 (“excellent”)]. Beta estimates can be interpreted as difference in mean adjusted AHEI component score for a 1-unit change in SRD (i.e., from “poor” to “fair,” “fair” to “good,” etc.)*.

Although not significant, as AHEI decreased, SRD was higher for the negatively scored components of red meats, SSBs, and sodium (adjusted mean AHEI component score difference by SRD, Table 4); beta estimates can be interpreted as the difference in mean adjusted AHEI component score between a given SRD category and the reference (“poor” SRD). Furthermore, as AHEI score increased, SRD was higher for vegetables, nuts and legumes, whole grains, alcohol, PUFA, and LCFA (Table 4). We observed similar patterns when intakes (servings, micrograms, or g/day) were modeled per food category in relation to mean adjusted AHEI (data not shown).

### Exploratory Analysis: SRD by Life Stage

Exploratory analyses of recalled SRD from previous life stages showed that recall of higher diet quality in earlier life stages (childhood and young adulthood) was associated with lower adjusted mean AHEI in adulthood. Mean adjusted AHEI was significantly lower among those who recalled SRD as “excellent” or “very good,” vs. “poor,” in young adulthood (β = −6.46, CI = −12.5, −0.43; β = −8.55, CI = −14.2, −2.92; Table 2). Similar results were observed with a three-point SRD scale (Supplementary Table 1).

### Sensitivity and Specificity

Sensitivity of SRD, against median AHEI score, was 59.7%, 95% CI (51.0, 68.3), while specificity was 34.2%, 95% CI (25.8, 42.5) (data not shown). Results closer to 100% indicate, for sensitivity, the ability of the SRD item to correctly identify those participants with higher AHEI scores and thereby better diets, and for specificity, results closer to 100% indicate the ability to identify those with lower AHEI scores indicating a truly poorer diet.

## Discussion

Our findings do not support the use of a single-item SRD measure as a valid assessment of current diet quality for adults in PR, except for the fruit and LCFA components of the AHEI. This is especially the case when distinguishing extreme SRD responses (e.g., excellent vs. poor), as is supported by previous studies reporting the relationship between Healthy Eating Index (HEI) score and SRD in other settings (4, 32, 33). We are aware of no study which has yet supported using a self-rated (perceived) diet quality measure in place of a measured diet quality tool (4).

Compared with previous studies, lower correlation between measured and perceived diet quality in the current study could reflect geographic, environmental, cultural, psychosocial, and socioeconomic differences influencing food access, availability, and familiarity. These differences may also be due to differences in sampling methods. The current study recruited visitors and patients from community clinics in San Juan, PR, while a study in Los Angeles (33) predominantly targeted socioeconomically disadvantaged participants. Approximately half of the participants in the current study reported < $10,000 annual income (about half of the median household income in PR); a similar proportion reported less than high school education in the Los Angeles study (33). However, the level of college education was higher than 50%. Alternatively, in a NYC study sampled *via* random phone calls, approximately half reported being white and/or college educated (4). These socioeconomic disparities in sampling could have manifested as differences in nutritional awareness and health literacy, both of which have been positively associated with HEI-2010 score (34). Nonetheless, we were able to adjust for some of the most cited factors related to demographics, socioeconomic status, and health behavior. We also examined waist circumference as a potential anthropometric confounder; however, the lack of significant association with SRD suggests that it may not act as such.

Differences in measurement also must be noted. The Mexican study (32) measured diet quality with the Mexican Diet Quality Index (MxDQI) score, based on 24-h recall; the NYC study (4) used the HEI-2010 score (Healthy Eating Index; similar to the AHEI), based on 24-h recall; and the Los Angeles study (33) was *via* multi-item measure, with particular emphasis on consumption of sugar-sweetened beverages and energy-dense snacks. The current study measured diet quality *via* AHEI score with culturally adapted FFQ data. The AHEI score has been shown to be strongly associated with cardiometabolic disease in PR (35); therefore, this is a strength in our design. While this survey presents challenges in terms of recall and portion estimation, this adapted FFQ has been validated for evaluating longer-term dietary patterns (36). Alternatively, 24-h recall measures present single-day mean estimates, which may not be reflective of usual patterns (37).

Most participants in the current study rated their diet positively (e.g., “good,” “very good,” or “excellent”). Similar results were observed among adults in Mexico (32), as well as among predominantly Latino adults of Los Angeles (33). This may be a consequence of social desirability (38, 39). Alternatively, nutritional awareness may play a role in how individuals respond (40). However, sensitivity analyses did not support this in the context of this study. Finally, this positive perception may be a consequence of our predominantly female sample, as gendered differences have been observed in the reporting of self-rated health and dietary intake (41–43). Future studies on the evaluation of environmental, cultural, psychosocial, and socioeconomic mechanisms underlying the intersection of perception, intention, and actual intake are warranted (44, 45).

This study also highlights the need for further research to examine which diet components participants consider when rating their own diets. While most component scores trended in the hypothesized direction, the only significant association with SRD was noted for fruit. In general, fruit and vegetables have been correlated with perceptions of healthfulness (46–49) and self-rated eating habits (33). A previous study in this same cohort showed that individuals consuming local fruit and vegetable more frequently (vs. never or rarely) had higher overall diet quality (50). Thus, it is possible that people place a higher health value on these two components, compared with the others. While most previous studies have combined fruit and vegetable consumption as one construct (32, 46), we separated them in relation to diet quality. Therefore, our observation that fruit, but not vegetables, is associated with perception of diet quality may be an indication that, in this PR context, fruit consumption is more linked with perception of diet quality than vegetable consumption. Local food environment characteristics and availability (51), specific to PR, may contribute to this distinction. Those who reported “excellent” vs. “poor” adulthood SRD exhibited significantly higher intake of fruit and/or LCFA. In line with dietary recommendations, similar results were reported in previous studies of diet components and either self-rated diet or health (5, 32, 52, 53). These observations raise the question of whether certain components hold more weight in an individual's estimation or perception of SRD. This has meaningful implications for the design and implementation of nutrition communication campaigns and educational interventions, to focus on meaningful targets for behavioral intervention and psychological change.

Our study is not without limitations. Our sample size was relatively small, which reduced statistical power to observe associations. Future research with larger sample sizes may improve interpretation of moderate scores, sitting between extreme options. Our cross-sectional design also limited our ability to assess causal direction between independent and dependent variables. Furthermore, we enlisted a convenience sample within San Juan metro area clinics, predominantly of women of older age reporting urban residency, limiting the generalizability of our sample.

Nevertheless, our study is one of the first to evaluate and characterize perceptions of diet quality in relation to dietary intake patterns in adults living in PR—with striking disparities related to diet access and health, especially with regard to cardiovascular health (8, 10). As many as 20% of health center patients have been reported to exhibit poor diet quality in PR (10). Differences in diet quality have been observed between residents of PR vs. those of the contiguous US, with those in PR exhibiting significantly lower average overall, as well as lower component scores (8, 10). This serves as impetus for future research on the relationship between food environment, culture, and diet quality.

Our study is also one of the first to adopt a life course approach in assessing SRD and measured intake. We recognize that there are several confounders in the relationship between past diet quality recall and current consumption. Our exploratory analysis poses new insights into how past diet perceptions may influence intake at a later life stage; these potential associations as well as the validity of the tools to assess past diet should be further explored. In contrast to expectation, diet quality perception in earlier life stages, especially in young adulthood, was inversely associated with higher currently measured diet quality. This does not necessarily imply that these individuals have changed their diet over time; however, this implies that people often consider their current diet quality to differ from their diet quality in previous life stages. It may be possible that those with higher AHEI score in adulthood perceived a less healthy diet quality in earlier life stages. How people perceive—or intend—for their diet quality to change over time may be influenced by socioeconomic status and health behavior, through increased risk and manifestation of chronic disease over the life course (54).

Overall, our research suggests that a single-item SRD measure may not be a particularly useful tool to measure actual diet quality for adults in PR. Larger studies should evaluate the possible use of the construct to distinguish extreme diet quality categories. The unexpected significant inverse association with earlier perceived diet is interesting; further investigation is required to understand how PR adults perceive diet quality and which factors may affect changes in perception of or actual dietary behavior over the life course. Before this single-item measure is used further with Latino/a adults in PR, additional research is needed regarding the mechanisms driving the relationship between perception in earlier life stages and current dietary habits, choices, and patterns in adulthood. Additionally, research is needed on the mechanisms underlying the cultural susceptibility of this measure, to better understand the sociocultural factors that impact choices around responses. In conclusion, a single-item SRD question has limited the application in capturing diet quality among adults in PR. Further research should resolve the limitations of this study before considering its use as a diet quality construct in research or clinical settings.

## Data Availability Statement

The raw data supporting the conclusions of this article will be made available by the authors, without undue reservation.

## Ethics Statement

The study was approved by Institutional Review Boards at Harvard T.H. Chan School of Public Health, Ponce Health Sciences University in PR, University of Massachusetts Lowell, and Northeastern University. Written informed consent was obtained from all participants. The patients/participants provided their written informed consent to participate in this study.

## Author Contributions

CG drafted the analytic plan, executed the analysis, and drafted the manuscript. AL-C, JO'N, and MT aided in the development of the research question, critically reviewed the analytic plan, aided in the interpretation of results, and critically revised the manuscript. KT, JO'N, and JM were responsible for the study design and interpretation of results and for the critical review of the manuscript. JM was also responsible for supervision of the analysis. All authors contributed to the article and approved the submitted version.

## Conflict of Interest

The authors declare that the research was conducted in the absence of any commercial or financial relationships that could be construed as a potential conflict of interest.
