# Supplementary material for: Association of a Single-Item Self-Rated Diet Construct With Diet Quality Measured With the Alternate Healthy Eating Index
Source: Front Nutr. 2021 May 5;8:646694. doi: 10.3389/fnut.2021.646694 (PMC8131508; doi:10.3389/fnut.2021.646694)
Supplement: Supplementary file 1 [file Table_1.pdf]

Supplementary Material

Supplemental Table 1. Mean Alternate Healthy Eating Index (AHEI) Score by Self-Rated Diet (SRD) at Three Life Stages (Adulthood, Young Adulthood, and Childhood) (n=247)

Life stage	SRD	Adjusted Mean AHEI (SE) [A]	B (95% CI) [B]	P-value [B]
Adulthood (current)	Excellent or very good (n=92, 37%)	60.5 (6.18)	2.21 (-1.49, 5.90)	0.24
	Good (n=87, 35%)	59.17 (4.48)	0.85 (-2.88, 4.58)	0.65
	Fair or poor (n=68, 28%)	58.3 (4.50)	Ref	Ref
Young adulthood (18-30y)	Excellent or very good (n=74, 30%)	54.7 (4.47)	-6.19 (-9.77, -2.61)	<0.001
	Good (n=83, 34%)	58.6 (4.42)	-2.27 (-5.70, 1.17)	0.19
	Fair or poor (n=90, 36%)	60.9 (4.36)	Ref	Ref
Childhood (<18y)	Excellent or very good (n=106, 43%)	58.6 (4.40)	-3.14 (-6.70, 0.435)	0.09
	Good (n=75, 30%)	56.8 (4.49)	-4.91 (-8.69, -1.13)	0.011
	Fair or poor (n=66, 27%)	61.7 (4.52)	Ref	Ref

AHEI = Alternate Healthy Eating Index (possible range=0.0-110.0, observed range=35.9-97.0, low to high diet quality).

SRD = Self-rated diet (possible range = 1-3, 'poor' or 'fair' to 'very good' or 'excellent' diet quality).

All regression models adjust for age, sex, ethnicity (Puerto Rican vs. other), education (less than 8th grade, high school or GED, or some college or more), income ($0-10,000; $10,001-$20,000; > $20,000), residency (i.e., urban vs. rural), marital status (married or with a partner vs. other), sleep duration (i.e., healthy, extreme, or missing), smoking status (i.e., current, never, past), and physical activity (i.e., sedentary, light, moderate, or missing).

[A] Shown as the adjusted mean (SE). Significance of differences in unadjusted mean values across SRD categories were also examined using ANOVA; differences were not found to be significant.

[B] SRD is modeled as a three-point categorical exposure (i.e., 'excellent or very good', 'good', and 'fair or poor'). The reference category is 'fair or poor.' GLM was used to evaluate the relationship between SRD in relation to continuous overall AHEI score. Beta estimates can be interpreted as difference in mean adjusted AHEI score between a given SRD category and the reference ('fair or poor').

Supplemental Table 2. Correlation Between Alternate Healthy Eating Index Scores and Self-Rated Diet at Three Life Stages (Adulthood, Young Adulthood, Childhood) (n=247)

	Mean (SD) [Range]		Rho (p-value)	
Scores	Overall [A] (n=247)	Adulthood [B]	Young Adulthood [B]	Childhood [B]
AHEI	60.1 (11.4) [35.9-97.0]	0.067 (0.29)	-0.236 (<0.001)***	-0.075 (0.24)
Vegetables	6.09 (2.90) [0.59-10]	0.094 (0.14)	-0.124 (0.051)*	-0.093 (0.14)
Fruits	2.95 (2.60) [0.01-10]	0.095 (0.14)	-0.091 (0.15)	0.022 (0.73)
Nuts and legumes	6.39 (3.22) [0.00-10.0]	-0.011 (0.87)	0.080 (0.209)	-0.022 (0.73)
Whole grains	5.36 (3.54) [0.00-10.0]	0.076 (0.23)	-0.134 (0.035)*	-0.057 (0.37)
Red meats	4.40 (3.46) [0.00-10.0]	-0.017 (0.80)	-0.207 (0.001)***	-0.105 (0.10)
SSB	1.64 (3.03) [0.00-10.0]	-0.056 (0.38)	-2.62 (<0.001)***	-0.06 (0.33)
Alcohol	6.25 (1.79) [0.00-10.0]	0.034 (0.60)	0.107 (0.092)	0.039 (0.54)
PUFA	6.12 (2.21) [0.95-10]	0.036 (0.57)	-0.005 (0.94)	0.009 (0.89)
Trans fats	7.92 (1.32) [1.66-10]	0.004 (0.95)	-0.188 (0.03)**	-0.049 (0.45)
Long-chain fatty acids	6.83 (2.52) [1.54-10]	0.055 (0.39)	0.029 (0.65)	0.017 (0.79)
Sodium	6.12 (3.21)[0-10]	-0.014 (0.83)	-0.118 (0.06)	0.036 (0.57)

SSB = sugar-sweetened beverages. PUFA = polyunsaturated fats.
AHEI = Alternate Healthy Eating Index (possible range for overall score=0.0-110.0, observed range=35.9-97.0, low to high diet quality; possible range for component scores=0.0-10.0).
Significance shown as * P <= 0.05; ** P <= 0.01; *** P <= 0.001.
SRD = Self-rated diet (possible range = 1-3, ‘poor’ or ‘fair’ to ‘very good’ or ‘excellent’ diet quality).
[A] Shown as the unadjusted mean AHEI score (SD) [range].
[B] Shown as Spearman’s Rho (p-value), interpreted as the direction and magnitude of correlation shared by SRD (3-point) and continuous AHEI component scores.

Supplemental Table 3. Adjusted Mean Waist Circumference by Self-Rated Diet as Categorical Variable, Among a Subset of Participants (n=202)

SRD	Adjusted mean WC (SE) [A]	B (95% CI) [B]	P-value [B]
**Excellent (n=30, 14.9%)**	91.2 (7.89)	5.32 (-7.69, 18.3)	0.42
**Very good (n=44, 21.8%)**	89.4 (8.35)	3.52 (-9.25, 16.3)	0.59
**Good (n=69, 34.2%)**	93.1 (7.62)	7.21 (-5.08, 19.5)	0.25
**Fair (n=50, 24.8%)**	94.5 (7.60)	8.57 (-3.95, 21.1)	0.18
**Poor (n=9, 4.46%)**	85.9 (9.37)	Ref	Ref

AHEI = Alternate Healthy Eating Index (possible range=0.0-110.0, observed range=35.9-97.0, low to high diet quality).
SRD = Self-rated diet (possible range = 1-5, 'poor', 'fair', 'good', 'very good', 'excellent' diet quality).
WC = Waist circumference in centimeters.
All regression models adjusted for age, sex, ethnicity (Puerto Rican vs. other), education (less than 8th grade, high school or GED, or some college or more), income ($0-10,000; $10,001-$20,000; > $20,000), residency (i.e., urban vs. rural), marital status (married or with a partner vs. other), sleep quantity (i.e., healthy, extreme, or missing), smoking status (i.e., current, never, past), and physical activity (i.e., sedentary, light, moderate, or missing).
[A] Shown as the adjusted mean (SE) in centimeters.
[B] SRD was modeled as a five-point categorical exposure (i.e., 'poor', 'fair', 'good', 'very good', 'excellent'). The reference category was 'poor.' GLM was used to evaluate the relationship between SRD in relation to continuous overall WC score. Beta estimates can be interpreted as difference in mean adjusted AHEI score between a given SRD category and the reference ('poor').

Supplemental Table 4. Mean Component Alternate Healthy Eating Scores by Self-Rated Diet Quality (n=247)

Components (range: 0-10)	Adulthood SRD: How would you describe your current diet? Unadjusted mean score (n=247)	Excellent or very good [A,B] (n=92)	Good [A,B] (n=87)	Fair or poor [A,B] (n= 68)	B (95% CI) [C]	p-value [C]
Vegetables	6.09 (2.90)	7.21 (1.20)	6.68 (1.16)	6.31 (1.16)	0.457 (-0.018,0.931)	0.06
Fruits	2.95 (2.60)	3.74 (1.05)	3.19 (1.01)	2.98 (1.02)	0.391 (-0.024,0.805)	0.06
Nuts and legumes	6.39 (3.22)	6.18 (1.31)	6.63 (1.31)	6.47 (1.35)	-0.053 (-0.589, 0.484)	0.85
Whole grains	5.36 (3.54)	3.33 (1.40)	2.78 (1.41)	3.48 (1.45)	0.339 (-0.236,0.914)	0.25
Red meats	4.40 (3.46)	2.54 (1.41)	3.25 (1.36)	2.78 (1.37)	-0.157 (-0.717,0.402)	0.58
SSB	1.64 (3.03)	1.58 (1.22)	2.49 (1.23)	1.82 (1.27)	-0.301 (-0.804,0.202)	0.24
Alcohol	6.25 (1.79)	7.31 (0.657)	7.68 (0.660)	7.92 (0.680)	0.154 (-0.118,0.425)	0.27
PUFA	6.12 (2.21)	7.10 (0.883)	6.88 (0.887)	7.05 (0.914)	0.078 (-0.284,0.439)	0.67
Trans fats	7.92 (1.32)	7.80 (0.500)	7.72 (0.503)	7.87 (0.518)	0.074 (-0.131,0.279)	0.48
LCFA	6.83 (2.52)	7.82 (1.05)	7.77 (1.01)	7.25 (1.02)	0.273 (-0.143,0.688)	0.20
Sodium	6.11 (3.21)	4.60 (1.33)	4.97 (1.29)	4.84 (1.29)	-0.135 (-0.663,0.393)	0.62

SSB = sugar-sweetened beverages. PUFA = polyunsaturated fats.
SRD = Self-rated diet (possible range = 1-3, 'poor' or 'fair' to 'very good' or 'excellent' diet quality).
LCFA = Long-chain fatty acids
All regression models adjusted for age, sex, ethnicity, education, income, residency (i.e., urban vs. rural), marital status, sleep quantity (i.e., healthy vs. extreme), smoking status (i.e., current, never, past), and physical activity (i.e., sedentary, light, and moderate or vigorous).
Significance shown as * P <= 0.05.
[A] Shown as the unadjusted mean AHEI score (SD) [range] by categorical SRD.
[B] SRD was modeled as a categorical exposure (i.e., 1 ('fair' or 'poor'), 2 ('good'), 3 ('excellent' or 'very good')); only associations found to be significant are reported. Reference category is 'fair or poor.'

[C] SRD was modeled via GLM as a continuous exposure (i.e., 1 ('fair' or 'poor') to 3 ('excellent' or 'very good')). Beta estimates can be interpreted as difference in mean adjusted AHEI component score between a given SRD category and the reference ('fair' or 'poor' SRD).
